# No Outbreak of Vancomycin and Linezolid Resistance in Staphylococcal Pneumonia over a 10-Year Period

**DOI:** 10.1371/journal.pone.0138895

**Published:** 2015-09-23

**Authors:** Josef Yayan, Beniam Ghebremedhin, Kurt Rasche

**Affiliations:** 1 Witten/Herdecke University, Witten, Department of Internal Medicine, Division of Pulmonary, Allergy and Sleep Medicine, HELIOS Clinic Wuppertal, Germany; 2 Institute of Medical Laboratory Diagnostics, Center for Clinical and Translational Research, HELIOS Clinic Wuppertal, Germany; University of Birmingham, UNITED KINGDOM

## Abstract

**Background:**

Staphylococci can cause wound infections and community- and nosocomial-acquired pneumonia, among a range of illnesses. *Staphylococcus aureus* and methicillin-resistant *S*. *aureus* (MRSA) have been rapidly increasing as a cause of infections worldwide in recent decades. Numerous reports indicate that *S*. *aureus* and MRSA are becoming resistant to many antibiotics, which makes them very dangerous. Therefore, this study retrospectively investigated the resistance to antimicrobial agents in all hospitalized patients suffering from community- or nosocomial-acquired pneumonia due to *S*. *aureus* and MRSA.

**Methods:**

Information from the study groups suffering from either community- or nosocomial-acquired pneumonia caused by *S*. *aureus* or MRSA was gathered by searching records from 2004 to 2014 at the HELIOS Clinic Wuppertal, Witten/Herdecke University, Germany. The findings of antibiotic resistance were analyzed after the evaluation of susceptibility testing for *S*. *aureus* and MRSA.

**Results:**

Total of 147 patients (63.9%, 95% CI 57.5%–69.8%), mean age 67.9 ± 18.5 years, with pneumonia triggered by *S*. *aureus*, and 83 patients (36.1%, 95% CI 30.2%–42.5%), mean age 72.3 ± 13.8 years, with pneumonia due to MRSA. *S*. *aureus* and MRSA developed no resistance to vancomycin (*P* = 0.019 vs. < 0.0001, respectively) or linezolid (*P* = 0.342 vs. < 0.0001, respectively). MRSA (95.3%) and *S*. *aureus* (56.3%) showed a high resistance to penicillin. MRSA (87.7%) was also found to have a high antibiotic resistance against ß-lactam antibiotics, compared to *S*. *aureus* (9.6%). Furthermore, MRSA compared to *S*. *aureus*, respectively, had increased antibiotic resistance to ciprofloxacin (90.1% vs. 17.0%), cefazolin (89.7% vs. 10.2%), cefuroxime (89.0% vs. 9.1%), levofloxacin (88.2% vs. 18.4%), clindamycin (78.0% vs. 14.7%), and erythromycin (76.5% vs. 20.8%).

**Conclusion:**

No development of resistance was found to vancomycin and linezolid in patients with pneumonia caused by *S*. *aureus* and MRSA.

## Introduction

*Staphylococcus* species are commonly found as members of the skin microbiome or mucosae that typically constitute a biological barricade, without causing disease in healthy adults [1]. *S*. *aureus* can often be found in the nose, vagina, inguinal areas, and underarms in adults, and it can represent a source of infection in patients with weakened immune systems, diabetes, chronic skin diseases, and skin injuries [2]. The consequences of infection with *S*. *aureus* can be, among others, community- and nosocomial-acquired pneumonia [3,4,5]. *S*. *aureus* possess several virulence factor genes and thus it is able to evade the host immune system [6]. In addition, most *S*. *aureus* infections are inherently resistant to antibiotics from the ß-lactam group, such as penicillin, which often makes it difficult to treat them [7]. A particular risk in hospitals is a special strain of methicillin-resistant *S*. *aureus* (MRSA) which is a major infection threat [8]. This bacterial strain has acquired resistance to numerous antibiotics and can be a health risk to immunocompromised patients in hospitals or nursing homes [9]. To date, infections with MRSA have occurred mainly in hospitals, where it is transmitted from human to human [10]. In recent years, cases of these types of infections have increasingly been recorded outside of hospitals, in care facilities and nursing homes [11]. *S*. *aureus* in particular rapidly develops resistance to many antibiotics, so the time may come when it is entirely resistant to all antibiotics [12]. The worldwide incidence of patients with MRSA pneumonia is rising. Vancomycin in combination with rifampicin was the first antibiotic prescribed for treating MRSA pneumonia. However, in addition to the low efficacy of vancomycin, it has been reported that there is resistance to it by MRSA [13]. Currently, linezolid is recommended for treating MRSA nosocomial-acquired pneumonia. Meanwhile, the latest reports show that MRSA is becoming resistant to linezolid [14]. With this in mind, this study will also ultimately serve to identify the timing of the increasing development of antimicrobial resistance in *S*. *aureus* and MRSA over the last 10 years. For this purpose, all necessary data from inpatients with pneumonia caused by *S*. *aureus* and MRSA, in accordance with the International Statistical Classification of Diseases (ICD J15.2) [15,16], were gathered by searching the database of HELIOS Clinic Wuppertal at Witten/Herdecke University, Germany. The goal of this clinical investigation was to identify antibiotic resistance over a period of 10 years according to susceptibility testing in the tracheal or bronchial secretions and blood cultures of patients with *S*. *aureus* and MRSA. Susceptibility to commonly used antibiotics was compared in patients with *S*. *aureus* and MRSA. The antibiotic treatments were also analyzed in patients with pneumonia within this study period. The timely, correct choice of effective antibiotics to treat *S*. *aureus* and MRSA infections should relieve the discomfort of the patients sooner, shorten their hospital stays, and lower their mortality rates.

## Material and Methods

### Ethics statement

All personally identifiable details of the study population were removed from the data sets previous to examination. The Health Care Ethics Committee at Witten/Herdecke University in Witten, Germany, approved the present investigation and all experimental protocols. Due to the retrospective study design, the Health Care Ethics Committee at Witten/Herdecke University renounced the requirement for printed, notified agreement.

### Setting

This study was conducted at the Department of Internal Medicine, Division of Pulmonary, Allergy and Sleep Medicine at HELIOS Clinic Wuppertal, which is a hospital with two locations in the districts of Barmen and Elberfeld. It is the largest hospital in the region of Bergisch Land, which is a low-mountain-range region within the German state of North Rhine-Westphalia. It has 967 beds and 26 departments, and each year the hospital treats approximately 50,000 inpatients and 100,000 outpatients. The Bergisch Lung Center at HELIOS Clinic Wuppertal has 70 beds, with treatment options for up to 16 beds in the intensive care unit and 6 beds in the intermediate care unit. In this study, all patients with *S*. *aureus* and MRSA pneumonia, who were treated on the general wards of all departments, intensive care units, and infectious-disease settings, were included. The majority of patients with pneumonia were treated at the clinic’s Division of Pulmonary, Allergy and Sleep Medicine.

### Patients

The present quality-control observational study retrospectively investigated resistance to antimicrobial agents in all inpatients with identified community- or nosocomial-acquired pneumonia caused by *S*. *aureus* or MRSA. Two study groups were formed according to the pathogenic cause of the pneumonia. The first group consisted of cases caused by *S*. *aureus*, and the second group was cases caused by MRSA. For this purpose, all required data for this study were accumulated from the clinic’s database, covering the period from January 1, 2004, to August 12, 2014.

### Definition of pneumonia

Pneumonia is an acute infection of the lung and is usually caused by *S*. *aureus* or MRSA. Typical clinical symptoms of pneumonia include cough, chest pain, fever, and difficulty breathing. Acute *S*. *aureus* or MRSA community-acquired pneumonia is a serious contamination of the pulmonary tissue, picked up during communal interaction with the public, whereas *S*. *aureus* or MRSA nosocomial-acquired pneumonia develops during a stay in the hospital [16]. The identification of *S*. *aureus* and MRSA pneumonia is made by means of X-ray investigations and sputum cultures [17,18]. The specific criteria for the selection of the *S*. *aureus* and MRSA pneumonia patients were that all of the patients were hospitalized and, upon X-ray investigation, they displayed new areas of infection. Additionally, the participants had to exhibit more than two of the following clinical aliments: trouble breathing, fever over 38°C, sputum generation, cough, and leukocytosis (white platelet count ≥ 10,000/μL).

### Clinical specimens

Expectorations from the pharynx, trachea, and bronchi were acquired in different ways depending on the individual case; the most common methods were sputum collection, throat swabs, tracheal secretions, and bronchoalveolar lavage. The bronchoalveolar lavage was executed by means of fiberoptic bronchoscopy. To obtain bronchial secretions from the lungs, approximately 20 ml of isotonic sodium chloride solution was infused into the bronchial tubes after the patient was given local anesthetic. The solution was then aspirated with the bronchoscope once more. Recovered bronchial and tracheal secretions were deposited in three separate sterilized 40-ml sample containers (Argyle^TM^, Covidien Ltd, Neustadt an der Donau, Germany). Throat-smears were carried out using a commercially available throat swab (MEUS Srl, Piove di Sacco, Italy), turning the swab while lightly pressing along the pharynx of inpatients with supposed pneumonia. Sputum was collected in a 30-ml antiseptic phlegm container (Salivette®, SARSTEDT, Nümbrecht, Germany). Obtained phlegm was subjected to microscopic investigation, which was performed with Gram staining at 80–1,000× enlargement, with at least five fields of vision according to the criteria created by Bartlett [19].

### Blood cultures

Approximately 20 ml of blood was collected for the discovery of germs from the blood stream by puncturing a vein using a blood-collection needle. The blood was then injected into two special blood culture media from BD BACTEC™ Instrumented Blood Culture Systems (Becton Dickson, Heidelberg, Germany). The susceptibility of antibiotics against isolates gained from the samples of blood cultures were compared with those from tracheal and bronchial secretions, sputum, and throat swabs in patients with pneumonia caused by *S*. *aureus* and MRSA.

### Identification and antimicrobial susceptibility testing

*Staphylococcus* was identified based on the growth on Columbia blood agar and chocolate agar (Becton Dickinson, Heidelberg) after 18–48 hours at 37°C with the use of 5% carbon dioxide and MALDI-TOF-MS (Bruker, Bremen, Germany).

For staphylococcal isolates, inhibition zone diameter breakpoints were used in accordance with the Clinical and Laboratory Standards Institute (CLSI) 2004–2011 laboratory agreement guidelines [20] and then with the European Committee on Antimicrobial Susceptibility Testing (EUCAST) breakpoints for 2012–2014 [21] (Table 1).

**Table 1:** MIC breakpoints for *S*. *aureus* according to EUCAST and CLSI guidelines.

	EUCAST	CLSI
Antimicrobial	Sensitive ≤ / Resistant > (mg/L)	Sensitive ≤ / Resistant > (mg/L)
Oxacillin	2/2	2/2
Vancomycin	2/2	2/8
Teicoplanin	2/2	8/16
Linezolid	4/4	4/4

**Abbreviations:** CLSI: Clinical and Laboratory Standards Institute; EUCAST: European Committee on Antimicrobial Susceptibility Testing; MIC: minimum inhibitory concentration

The method of susceptibility testing for identifying staphylococci was executed by means of the disc diffusion method established by Kirby-Bauer [22]. In cases of discrepancies or insufficient readings, the assessment of the minimum inhibitory concentration (MIC) was performed utilizing an E-test for particular antimicrobials, and the results were interpreted according to the EUCAST criteria [21]. Intermediate isolates were grouped along with resistant isolates.

### Tested antibiotics

MRSA involves strains of the species *S*. *aureus* that are resistant to methicillin and that cause pneumonia. Most staphylococci are resistant to penicillins due to their production of penicillinase. Staphylococci are reported to be susceptible to penicillins and methicillin when they are negative for penicillinase and susceptible to methicillin in susceptibility testing. With very few exceptions, MRSA infections are resistant to all ß-lactam agents. *S*. *aureus* is mostly resistant to methicillin with oxacillin MIC values of > 2 mg/L. The susceptibility of staphylococci to cephalosporins and carbapenems is derived from the cefoxitin susceptibility. *S*. *aureus* is methicillin-resistant with cefoxitin MIC values of > 4 mg/L. The antibiotics examined in this study were penicillin, oxacillin, ampicillin-sulbactam, ampicillin, piperacillin, piperacillin-tazobactam, cefotaxime, cefazolin, cefepime, cefuroxime, tetracycline, levofloxacin, erythromycin, ciprofloxacin, co-trimoxazole, clindamycin, gentamicin, tobramycin, vancomycin, teicoplanin, linezolid, rifampicin, fosfomycin, and fusidic acid.

The antimicrobials that were the most frequently used for the treatment of pneumonia and that were the most examined in susceptibility testing were compared with the other antibiotics. The rate of usage of these antimicrobials in routine clinical practice for the care of hospitalized patients suffering from *S*. *aureus* and MRSA pneumonia was noted.

### Laboratory tests

The amount of C-reactive protein (CRP) from the blood plasma (< 6 mg/L) was evaluated using a 4.7-ml SARSTEDT Monovette®, including lithium heparin and the COBAS® 6000 analyzer series c 501 (F. Hoffmann-La Roche Ltd, Mannheim, Germany). The quantity of leukocytes (4,000–10,000/μL) in the blood was measured with a 2.7-ml EDTA Monovette® through laser-based biophysical technology and a hematology analyzer on the Sysmex® XE 2100 (Sysmex Company, Norderstedt, Germany).

### Comorbidities

Acute and chronic concomitant diseases were assessed in all inpatients suffering from *S*. *aureus* and MRSA pneumonia. Comorbidity was considered the presence of one or more supplementary illnesses at the same time as the main disease of pneumonia. In addition, the duration of the hospital stay was measured in inpatients with *S*. *aureus* and MRSA pneumonia.

The number of deaths during hospital stays was computed in the patients with *S*. *aureus* and MRSA pneumonia, and survival probabilities were calculated by means of the Kaplan-Meier method.

### Statistical analysis

The categorical numbers were stated as percentages, whereas continuous numbers were indicated as means and standard deviations. The statistical computations were completed at the 95% confidence interval (CI) used to calculate the sex differences of inpatients, specimens, species, and comorbidities, as well as the various means of acquiring pneumonia caused by *S*. *aureus* and MRSA. A statistical calculation using one-way analysis of variance (ANOVA) for free variables was carried out to evaluate each *S*. *aureus* and MRSA isolate, categorized as sensitive, intermediate, or resistant to the antibiotics, as well as the differences in the ages of the study population, laboratory tests, and duration of hospital stays between *S*. *aureus* and MRSA pneumonia patients. An odds ratio (OR) was calculated for the likelihood of sensitivity or resistance to antimicrobials used against *S*. *aureus* and MRSA. After assessing the susceptibility testing in patients with *S*. *aureus* or MRSA pneumonia, the antibiotic most frequently used for therapy and the antibiotic most examined in susceptibility testing were matched in to the resistance rate, by means of the OR, with the other antibiotic substances. For comparison, the antibiotic with the lowest rate of resistance was also evaluated by the OR with the other antibiotic substances assessed in the susceptibility testing. The OR was also used to compare gender differences, acquisition route of pneumonia, sampling methods, deaths, and comorbidities between patients with *S*. *aureus* or MRSA pneumonia. Comprehensive two-tailed examinations were completed, and statistical significance was expressed at a *P* value below 0.05.

## Results

In the HELIOS Clinic database from January 1, 2004, to August 12, 2014, a total of 6,932 patients of all ages were identified with pneumonia caused by various microorganisms. There were 358 patients with staphylococcal pneumonia (ICD J15.2). Of these, 97 patients were excluded because either we did not have the right to access their records from the Clinic of Neurology and Clinical Neurophysiology or they were under 18 years old and were hospitalized in the clinic’s Department of Pediatric and Adolescent Medicine. Thirty-one inpatients with pneumonia caused by other *Staphylococcus* species were also excluded. After excluding these groups, 230 patients with staphylococcal pneumonia met the inclusion criteria for this investigation. Of these, 147 patients (63.9%, 95% CI 57.5%–69.8%) with pneumonia triggered by *S*. *aureus* formed the first study group, and 83 patients (36.1%, 95% CI 30.2%–42.5%) with pneumonia due to MRSA formed the reference group. Both groups mainly consisted of older people, and the mean age was not statistically significantly increased in patients with MRSA pneumonia compared to patients with *S*. *aureus* pneumonia (Table 2). Similarly, the male sex was over-represented but without statistical significance in both groups (Table 2). While *S*. *aureus* was discovered more in patients with community-acquired pneumonia, MRSA caused slightly more cases of nosocomial-acquired pneumonia (Table 2). In both groups, isolates were most often detected in tracheal secretions (Table 2). In general, the number of patients with pneumonia due to *S*. *aureus* was higher than those with MRSA in this study population (Table 2).

**Table 2:** Comparison of gender, acquisition, and sampling in patients with pneumonia caused by *S*. *aureus* compared to MRSA.

	*S*. *aureus* (n = 147) (%)	MRSA (n = 83) (%)	Odds ratio (95% CI %), *P* value
**Age** mean ± SD (years)	67.9 ± 18.5	72.3 ± 13.8	0.058
**Gender**			
Male	89 (60.5)	57 (68.7)	0.7 (0.4–1.2), 0.220
Female	58 (39.5)	26 (31.3)	0.7 (0.4–1.2), 0.220
**Acquisition of pneumonia**			
Community-acquired pneumonia	67 (45.6)	32 (38.6)	1.3 (0.8–2.3), 0.302
Nosocomial-acquired pneumonia	58 (39.5)	35 (42.2)	0.9 (0.5–1.5), 0.687
Aspiration pneumonia	22 (15.0)	16 (19.3)	0.7 (0.4–1.5), 0.399
**Sampling**			
Tracheal secretion	66 (44.9)	52 (62.7)	0.5 (0.3–0.8), **0.01**
Bronchial secretion	48 (32.7)	14 (16.9)	2.4 (1.2–4.7), **0.011**
Venous blood culture	15 (10.2)	2 (2.4)	4.6 (1.0–20.6), **0.046**
Sputum	14 (9.5)	9 (10.8)	0.9 (0.4–2.1), 0.749
Arterial blood culture	3 (2.0)	1 (1.2)	1.7 (0.2–16.7), 0.645
Throat swab	1 (0.7)	3 (3.6)	0.2 (0.02–1.8), 0.144
Secretion drainage	0	2 (2.4)	0.1 (0–2.3), 0.157
**Deaths**	29 (19.7)	31 (37.3)	0.4 (0.2–0.8), **0.004**
**Duration of hospital stay** mean +SD (days)	18.6 ± 16.2	19.7 ± 18.5	0.632
**Laboratory tests**			
CRP (< 6 mg/L) mean + SD	98.5 ± 113.7	76.1 ± 80.1	0.112
Leukocytes (4,000–10,000/μL) mean + SD	12,217.2 ± 6,494.3	12,179.3 ± 6,242.6	1.0

**Abbreviations:** CI: confidence interval; CRP: C-reactive protein; SD: standard deviation; MRSA: methicillin-resistant *Staphylococcus aureus*. **Note:** Significant *P* values are shown in bold.

On the antibiograms for patients with *S*. *aureus* compared to MRSA pneumonia, respectively, 83.2 ± 47.3 vs. 30.3 ± 32.3 of the infections were categorized as sensitive, 4.7 ± 22.6 vs. 0.4 ± 1.6 as intermediate, and 19.5 ± 30.4 vs. 37.4 ± 33.0 as resistant to a specific antibiotic, which was significantly different between the groups (*P* < 0.0001; Table 3).

**Table 3:** Susceptibility to various antibiotics according to antibiogram testing and number of antibiotics administered for the treatment of pneumonia caused by *S*. *aureus* compared to MRSA.

		No. of antibiotics used		No. of antibiotics tested on antibiogram		Sensitive		Intermediate		Resistant		Odds ratio (95% CI), *P* value	Odds ratio (95% CI), *P* value compared with piperacillin + tazobactam		Odds ratio (95% CI), *P* value compared with vancomycin	
Drug groups	Active substance	*S*. *aureus*	MRSA	*S*. *aureus*	MRSA	*S*. *aureus*	MRSA	*S*. *aureus*	MRSA	*S*. *aureus*	MRSA	*S*. *aureus* vs. MRSA	*S*. *aureus*	MRSA	*S*. *aureus*	MRSA
Penicillins	Penicillin	0	0	143	81	37	1	0	0	106	80	27.9 (3.8–207.9), **0.001**	0.03 (0–0.1), **<0.0001**	0.1 (0–0.7), **0.023**	0.001 (0–0.02), **<0.0001**	0.0001 (0–0.003), **<0.0001**
	Oxacillin	1	0	144	82	131	9	1	1	12	72	87.3 (35.1–217.2), **<0.0001**	1.1 (0.5–2.5), 0.849	0.9 (0.3–2.3), 0.807	0.04 (0–0.6), **0.022**	0.0008 (0–0.01), **<0.0001**
	Ampicillin	0	0	141	82	37	2	0	0	104	80	14.2 (3.3–60.8), **0.0003**	0.3 (0–0.1), **<0.0001**	0.2 (0–0.8), **0.029**	0.001 (0–0.02), **<0.0001**	0.0002 (0–0.004), **<0.0001**
	Piperacillin	2	0	92	54	21	1	0	0	71	53	15.7 (2.0–120.2), **0.008**	0.03 (0–0.1), **<0.0001**	2.1 (0.9–4.8), 0.077	0.001 (0–0.02), **<0.0001**	0.0002 (0–0.004), **<0.0001**
Penicillin + ß-lactamase inhibitors	Ampicillin + sulbactam	22	7	126	82	113	10	0	0	13	72	62.6 (26.1–150.3), **<0.0001**	0.9 (0.4–1.9), 0.720	1.0 (0.4–2.5), 0.977	0.03 (0–0.5), **0.015**	0.0009 (0–0.02), **<0.0001**
	Piperacillin + tazobactam	84	43	144	81	131	10	0	0	13	71	71.5 (29.9–171.4), **<0.0001**	0.03 (0–0.6), **0.019**	0.0009 (0–0.02), **<0.0002**		
Cephalosporins	Cefuroxime	29	9	143	82	130	9	0	0	13	73	81.1 (33.1–198.9), **<0.0001**	1.0 (0.4–2.2), 0.985	0.9 (0.3–2.3), 0.785	0.03 (0–0.6), **0.019**	0.0008 (0–0.01), **<0.0001**
	Cefotaxime	1	0	39	35	35	3	0	0	4	32	93.3 (19.4–449.5), **0.0001**	0.9 (0.3–2.8), 0.815	0.7 (0.2–2.6), 0.556	0.03 (0–0.5), **0.017**	0.0007 (0–0.01), **< 0.0001**
	Cefazolin	2	0	127	68	114	7	0	0	13	61	76.4 (29.0–201.6), **<0.0001**	0.9 (0.4–2.0), 0.736	0.8 (0.3–2.3), 0.695	0.03 (0–0.5), **0.015**	0.0007 (0–0.01), **<0.0001**
	Cefepime	1	0	30	19	29	1	0	0	1	18	522.0 (30.7–8877.1), **<0.0001**	2.9 (0.4–22.9), 0.318	0.4 (0–3.3), 0.390	0.1 (0–1.7), 0.103	0.0005 (0–0.01), **<0.0001**
Tetracycline	Tetracycline	0	0	59	69	59	68	0	0	0	1	2.6 (0.1–65.2), 0.560	12.2 (0.7–208.9), 0.084	482.8 (60.2–3873.8), **<0.0001**	0.4 (0–21.1), 0.661	0.3 (0–7.0), 0.438
Gyrase inhibitors	Ciprofloxacin	12	3	141	81	6	0	111	8	24	73	39.0 (2.1–717.8), **0.014**	0.2 (0–0.1), **<0.0001**	0.05 (0–0.8), **0.035**	0.001 (0–0.017), **<0.0001**	0 (0–0.002), **<0.0001**
	Levofloxacin	6	2	103	68	84	7	0	1	19	60	37.9 (15.0–95.8), **< 0.0001**	0.4 (0.2–0.9), **0.033**	0.8 (0.3–2.3), 0.719	0.02 (0–03), **0.004**	0.0008 (0–0.01), **< 0.0001**
Macrolide	Erythromycin	7	3	144	81	113	19	1	0	30	62	12.3 (6.4–23.6), **<0.0001**	0.4 (0.2–0.8), **0.006**	2.2 (0.9–5.0), 0.069	0.01 (0–0.2), **0.002**	0.002 (0–0.03), **<0.0001**
Trimethoprim + Sulfonamide	Co-trimoxazole	2	0	143	81	140	81	0	0	3	0	0.2 (0–4.8), 0.356	4.6 (1.3–16.6), **0.019**	129.0 (63.9–19281.1), **<0.0001**	0.1 (0–2.7), 0.195	1.0 (0.02–51.0), 1.0
Licosamide	Clindamycin	4	0	143	82	122	18	0	0	21	64	20.7 (10.3–41.5), **<0.0001**	0.6 (0.3–1.2), 0.142	2.0 (0.9–4.6), 0.108	0.02 (0–0.3), **0.006**	0.002 (0–0.03), **<0.0001**
Aminoglycosides	Gentamicin	6	4	145	81	137	79	0	0	8	2	0.4 (0.1–2.1), 0.298	1.7 (0.7–4.2), 0.255	280.5 (59.4–1323.5), **<0.0001**	0.1 (0–1.0), **0.049**	0.2 (0.01–4.1), 0.294
	Tobramycin	0	0	55	34	45	18	0	0	10	16	4.0 (1.5–10.5), **0.0047**	0.4 (0.2–1.1), 0.076	8.0 (3.1–20.5), **<0.0001**	0.02 (0–0.3), **0.004**	0.01 (0–0.12), **0.0006**
Glycopeptide	Vancomycin	14	40	143	81	143	81	0	0	0	0	1.8 (0–89.6), 0.778	29.5 (1.7–500.6), **0.019**	1110.0 (63.9–19281.1), <0.0001		
	Teicoplanin	0	0	29	20	29	20	0	0	0	0	1.4 (0–75.5), 0.857	6.1 (0.4–104.8), 0.216	279.2 (15.7–4969.9), **0.0001**	0.2 (0–10.6), 0.431	0.3 (0.005–13.1), 0.493
Oxazolidinone	Linezolid	1	6	19	64	19	64	0	0	0	0	0.3 (0–15.7), 0.553	4.0 (0.2–70.1), 0.342	878.4 (50.5–15292.5), **<0.0001**	0.1 (0–7.0), 0.322	0.8 (0.02–40.4), 0.907
Rifamycin	Rifampicin	4	25	120	79	120	77	0	0	0	2	7.8 (0.4–164.1), 0.188	24.7 (1.5–420.7), **0.027**	273.4 (57.9–1290.6), **<0.0001**	0.8 (0–42.6), 0.931	0.2 (0.01–4.0), 0.287
Epoxide	Fosfomycin	0	1	114	76	112	70	0	0	2	6	4.8 (0.9–24.4), 0.059	5.6 (1.2–25.2), **0.026**	82.8 (28.6–240.2), **<0.0001**	0.2 (0–3.3), 0.233	0.007 (0.004–1.2), 0.067
Fusidic acid	Fusidic acid	0	0	91	73	90	73	0	0	1	0	0.4 (0–10.2), 0.587	8.9 (1.1–69.5), **0.037**	1001.0 (57.6–17404.1), **<0.0001**	0.2 (0–5.2), 0.341	0.9 (0.002–46.0), 0.959

**Abbreviations:** CI: confidence interval; MRSA: methicillin-resistant *Staphylococcus aureus*. **Note:** Statistically significant *P* values are shown in bold.

Piperacillin-tazobactam was the most frequently administered antibiotic for the treatment of patients with *S*. *aureus* or MRSA pneumonia in this investigation, but there were differences in the second and third most frequently used antibiotics between the groups (Table 3). Cefuroxime was the second most used antibiotic for the treatment of *S*. *aureus* pneumonia, and vancomycin was the second most used for MRSA pneumonia. Finally, ampicillin-sulbactam was the third most frequently administered antimicrobial agent for the treatment of *S*. *aureus* pneumonia, and rifampicin was the third most used for MRSA pneumonia (Table 3). Simultaneous antibiotic resistance in both groups showed that *S*. *aureus* compared to MRSA developed no resistance to vancomycin (*P* = 0.019 vs. < 0.0001) or linezolid (*P* = 0.342 vs. < 0.0001) (Table 2). While MRSA (95.3%) mostly developed a high resistance to penicillins, *S*. *aureus* (56.3%) had significantly less development of resistance to penicillins (Table 3). A similar result of high antibiotic resistance was also found for ß-lactam antibiotics; MRSA (87.7%) showed a high resistance, and *S*. *aureus* (9.6%) had a much lower resistance rate to these agents (Table 3). The same observations were found with other antibiotics. MRSA compared to *S*. *aureus*, respectively, had high antibiotic resistance to ciprofloxacin (90.1% vs. 17.0%), cefazolin (89.7% vs. 10.2%), cefuroxime (89.0% vs. 9.1%), levofloxacin (88.2% vs. 18.4%), clindamycin (78.0% vs. 14.7%), and erythromycin (76.5% vs. 20.8%) (Table 3). Apart from the detection of small amounts of isolates in blood cultures in 18 (12.2%) patients with *S*. *aureus* pneumonia and in 3 (3.6%) patients with MRSA pneumonia (Table 4), *S*. *aureus* and MRSA showed high antibiotic resistance against penicillins and less resistance to other antibiotics (Table 4). In general, the resistance rate of isolates in blood cultures appeared to be a little higher than in expectorates, especially to penicillins and ß-lactam antibiotics (Table 4). However, the number of cases of the isolates from blood cultures from patients with pneumonia caused by *S*. *aureus* or MRSA was very low, making a clear statement about the susceptibility of these isolates against antimicrobial agents.

**Table 4:** Comparison of the susceptibility of antibiotics against isolates extracted from blood cultures with those from tracheal and bronchial sputum secretions and throat swabs in patients with pneumonia caused by *S*. *aureus* and MRSA.

		Expectorations *S*. *aureus* (n = 129), MRSA (n = 78), Blood cultures *S*. *aureus* (n = 18), MRSA (n = 3)							Compared expectoration + blood cultures	
			Sensitive		Intermediate		Resistant		Odds ratio (95% CI), *P* value	Odds ratio (95% CI), *P* value
Drug group	Active substance	Sampling	*S*. *aureus*	MRSA	*S*. *aureus*	MRSA	*S*. *aureus*	MRSA	*S*. *aureus*	MRSA
Penicillins	Penicillin	Expectorations	31	1	0	0	94	75	0.7 (0.2–1.9), 0.442	0.1 (0–4.1), 0.252
		Blood cultures	6	0	0	0	12	3		
	Oxacillin	Expectorations	113	9	1	1	12	68	0.2 (0.01–4.3), 0.337	0.7 (0.03–15.6), 0.818
		Blood cultures	18	0	0	0	0	2		
	Ampicillin	Expectorations	32	2	0	0	91	75	0.9 (0.3–2.8), 0.874	0.2 (0.01–5.8), 0.374
		Blood cultures	5	0	0	0	13	3		
	Piperacillin	Expectorations	17	1	0	0	59	49	0.9 (0.2–3.0), 0.820	0.2 (0–4.7), 0.283
		Blood cultures	4	0	0	0	12	2		
Penicillin + ß-lactamase inhibitors	Ampicillin + sulbactam	Expectorations	98	10	0	0	13	67	0.2 (0.01–4.2), 0.324	1.1 (0.1–22.6), 0.956
		Blood cultures	15	0	0	0	0	3		
	Piperacillin + tazobactam	Expectorations	113	10	0	0	13	66	0.2 (0.01–4.0), 0.311	1.1 (0.1–23.0), 0.948
		Blood cultures	18	0	0	0	0	3		
Cephalosporins	Cefuroxime	Expectorations	112	9	0	0	13	68	0.2 (0.01–4.0), 0.308	1.0 (0.05–20.3), 0.985
		Blood cultures	18	0	0	0	0	3		
	Cefotaxime	Expectorations	28	3	0	0	4	27	0.4 (0.02–8.7), 0.577	0.9 (0.04–21.1), 0.943
		Blood cultures	7	0	0	0	0	3		
	Cefazolin	Expectorations	101	7	0	0	13	57	0.3 (0.02–5.0), 0.384	0.7 (0.03–14.9), 0.789
		Blood cultures	13	0	0	0	0	2		
	Cefepime	Expectorations	18	1	0	0	1	15	0.5 (0.02–14.3), 0.710	0.5 (0.02–15.5), 0.682
		Blood cultures	11	0	0	0	0	2		
Tetracycline	Tetracycline	Expectorations	52	63	0	0	0	1	7.0 (0.1–379.9), 0.340	6.4 (0.2–177.0), 0.296
		Blood cultures	7	3	0	0	0	0		
Gyrase inhibitors	Ciprofloxacin	Expectorations	3	0	98	8	22	69	0.1 (0.01–0.8), **0.029**	0.04 (0–2.2), 0.114
		Blood cultures	3	0	13	0	2	2		
	Levofloxacin	Expectorations	75	7	0	1	18	55	0.5 (0.06–3.9), 0.478	0.9 (0.04–20.2), 0.972
		Blood cultures	9	0	0	0	1	3		
Macrolide	Erythromycin	Expectorations	101	19	1	0	24	57	2.1 (0.7–6.2), 0.176	2.4 (0.1–48.1), 0.573
		Blood cultures	12	0	0	0	6	3		
Trimethoprim + sulfonamide	Co-trimoxazole	Expectorations	123	76	0	0	2	0	3.6 (0.3–42.1), 0.304	153.0 (6.6–3573.9), **0.002**
		Blood cultures	17	3	0	0	1	0		
Licosamide	Clindamycin	Expectorations	109	18	0	0	17	59	2.0 (0.6–6.8), 0.280	2.2 (0.1–44.1), 0.612
		Blood cultures	13	0	0	0	4	3		
Aminoglycosides	Gentamicin	Expectorations	120	74	0	0	7	2	1.0 (0.1–8.7), 0.994	4.3 (0.2–106.6), 0.378
		Blood cultures	17	3	0	0	1	0		
	Tobramycin	Expectorations	39	16	0	0	8	14	1.6 (0.3–9.6), 0.591	1.1 (0.1–20.0), 0.927
		Blood cultures	6	1	0	0	2	1		
Glycopeptide	Vancomycin	Expectorations	125	77	0	0	0	0	6.8 (0.1–352.4), 0.342	31.0 (0.5–1903.3), 0.102
		Blood cultures	18	2	0	0	0	0		
	Teicoplanin	Expectorations	22	16	0	0	0	0	3.0 (0.1–164.8), 0.591	6.6 (0.1–414.3), 0.372
		Blood cultures	7	2	0	0	0	0		
Oxazolidinone	Linezolid	Expectorations	18	59	0	0	0	0	12.3 (0.2–872.0), 0.248	17.0 (0.3–991.0), 0.172
		Blood cultures	1	3	0	0	0	0		
Rifamycin	Rifampicin	Expectorations	109	73	0	0	0	1	9.5 (0.2–502.9), 0.266	7.0 (0.2–204.7), 0.259
		Blood cultures	11	3	0	0	0	0		
Epoxide	Fosfomycin	Expectorations	100	65	0	0	2	6	1.6 (0.1–35.4), 0.763	1.4 (0.1–31.0), 0.816
		Blood cultures	12	3	0	0	0	0		
Fusidic acid	Fusidic acid	Expectorations	83	69	0	0	0	0	33.4 (1.2–893.6), **0.036**	27.8 (0.5–1708.0), 0.114
		Blood cultures	7	2	0	0	1	0		

**Abbreviations:** MRSA: methicillin-resistant *Staphylococcus aureus*, CI: confidence interval. **Note:** Statistically significant *P* values are shown in bold.

The mean level of CRP in the blood samples of hospitalized patients with *S*. *aureus* pneumonia was not significantly elevated compared to patients with pneumonia triggered by MRSA (Table 2). The same applied to leukocytes; their average number was not considerably higher in the plasma of patients with pneumonia due to *S*. *aureus* compared to those with MRSA (Table 2).

The most commonly found acute comorbidity was acute respiratory failure in both study groups (Table 5). The most frequent chronic concomitant disease in patients with pneumonia caused by *S*. *aureus* and MRSA was arterial hypertension (Table 5).

**Table 5:** Acute and chronic comorbidities of patients with pneumonia caused by *S*. *aureus* compared to MRSA. Comorbidities were only considered if they were more than 10% in one of the groups even if they were less than 10% in the other group.

Cardiovascular disease	*S*. *aureus* (n = 147) (%)	MRSA (n = 83) (%)	Odds ratio (95% CI, *P* value compared *S*. *aureus* and MRSA patients
Anemia	25 (17.0)	19 (22.9)	0.7 (0.4–1.3), 0.277
Aneurysm	12 (8.2)	9 (10.8)	0.7 (03–1.8), 0.499
Cardiac decompensation	20 (13.6)	15 (18.1)	0.7 (03–1.5), 0.367
Cardiac dysrhythmia	21 (14.3)	14 (16.9)	0.8 (0.4–1.7), 0.601
Coronary heart disease	49 (33.3)	21 (25.3)	1.5 (0.8–2.7), 0.205
Heart failure	31 (21.1)	20 (24.1)	0.8 (0.4–1.6), 0.598
High blood pressure	59 (40.1)	58 (69.9)	0.3 (0.2–0.5), **< 0.0001**
Myocardial infarction	18 (12.2)	8 (9.6)	1.3 (0.5–3.2), 0.550
Sepsis	14 (9.5)	9 (10.8)	0.9 (0.4–2.1), 0.749
State after heart attack	19 (12.9)	23 (27.7)	0.4 (0.2–0.8), **0.006**
**Pulmonary disease**			
Acute respiratory failure	34 (23.1)	22 (26.5)	0.8 (0.4–1.6), 0.567
Chronic obstructive lung disease	37 (25.2)	25 (30.1)	0.8 (0.4–1.4), 0.417
Long term oxygen therapy	17 (11.6)	9 (10.8)	1.1 (0.5–2.5), 0.868
Pleural effusion	16 (10.9)	5 (6.0)	1.9 (0.7–5.4), 0.226
Pulmonary emphysema	17 (11.6)	4 (4.8)	2.5 (0.8–8.0), 0.098
**Gastrointestinal disease**			
Diabetes	37 (25.2)	19 (22.9)	1.1 (0.6–2.1), 0.699
Hyperlipidemia	15 (10.2)	3 (3.6)	3.0 (0.9–10.8), 0.087
**Kidney disease**			
Acute renal failure	25 (17.0)	13 (15.7)	1.1 (0.5–2.3), 0.792
Acute urinary tract infection	15 (10.2)	13 (15.7)	0.6 (0.3–1.4), 0.227
Chronic renal failure	22 (15.0)	16 (19.3)	0.7 (0.4–1.5), 0.399
Electrolyte imbalance	20 (13.6)	5 (6.0)	2.5 (0.9–6.8), 0.084
**Neurologic disease**			
Epilepsy	15 (10.2)	14 (16.9)	0.6 (0.3–1.2), 0.148
State after stroke	22 (15.0)	16 (19.3)	0.7 (0.4–1.5), 0.399
**Psychiatric disease**			
Dementia	6 (4.1)	13 (15.7)	0.2 (0.1–0.6), **0.004**
Smoker	21 (14.3)	9 (10.8)	1.4 (0.6–3.1), 0.458

**Abbreviations:** MRSA: methicillin-resistant *Staphylococcus aureus*. **Note:** Statistically significant *P* values are shown in bold.

The length of hospital stay was not appreciably increased in patients with pneumonia due to MRSA compared to patients with *S*. *aureus* pneumonia (Table 2). There were significantly more deaths related to MRSA pneumonia compared to deaths related to *S*. *aureus* pneumonia (Table 2). The survival rate was 62.7% (95% CI 49.5%–75.8%) in hospitalized patients with MRSA, clearly reduced compared to patients with *S*. *aureus* pneumonia, whose survival rate was 80.3% (95% CI 73.1%–87.5%).

## Discussion

Community-acquired pneumonia is a serious inflammation of the lower airways [23], and it is frequently caused by *S*. *aureus* [24]. As demonstrated in this study, staphylococcal community-acquired pneumonia occurs more frequently than staphylococcal nosocomial-acquired pneumonia. *S*. *aureus* is also a common source of nosocomial-acquired pneumonia globally [25], and it has been established as a main source of infection causing nosocomial-acquired pneumonia [26]. Aspiration pneumonia is usually caused by microorganisms such as *S*. *aureus* that come from the nasopharyngeal area. Although little is known about the exact incidence of aspiration pneumonia [27], staphylococcal aspiration pneumonia was detected often in the present study. However, reports on the frequency of aspiration pneumonia differ greatly in the literature [28].

The incidence of pneumonia caused by MRSA varies by region and hospital, but there have been reports of rising rates in recent years [29]. Community- and nosocomial-acquired pneumonia caused by MRSA was also observed frequently in this study. Approximately 6.6 MRSA cases per 100,000 citizens occurred in 2011 in the German state of North Rhine-Westphalia. There are few epidemiologic figures for MRSA in this geographical region, and experts argue that they are difficult to compare. The number of patients with MRSA fell from 1,500 in 2010 to 1,100 in 2014 at HELIOS Clinic Wuppertal [30].

The results of the present ten-year observational study demonstrated that *S*. *aureus* and MRSA, as causative organisms of community- and nosocomial-acquired pneumonia, showed no development of resistance to vancomycin and linezolid in susceptibility tests in patients undergoing antibiotic treatment during the study period. It is noteworthy that an elevation trend in the susceptibility to vancomycin was observed over the past ten years but the number of cases of pneumonia due to *S*. *aureus* and MRSA also increased significantly in the same period covered by the present study, and the MIC breakpoints for vancomycin were different after switching from the CLSI criteria the EUCAST guidelines in 2011. Vancomycin was used for the management of serious staphylococcal pneumonia, but it has been replaced in clinical management on the grounds of efficacy and toxicity in favor of other novel antibiotics [31]. The arrival of pseudomembranous enterocolitis and an increase in MRSA resulted in the revival of using vancomycin as a reserve antibiotic in the management of serious staphylococcal pneumonia in clinical practice [31]. However, concerns arose almost immediately about the therapeutic benefit of this antibiotic. Resistance to vancomycin emerged first in *Enterococcus* and then in *Staphylococcus* [32,33], and different varieties of antibiotic resistance to vancomycin have been recognized since then [34]. Due to the increasing emergence of resistance to vancomycin, the need arises for alternative therapies. New treatment options for infections with MRSA include linezolid [35].

Only Gram-positive pathogens are affected by the antibiotic effects of linezolid. In the presence of vancomycin resistance, linezolid is the reserve antibiotic of choice [36]. Many studies have examined linezolid and vancomycin in the treatment of Gram-positive bacterial infectious diseases. One study demonstrated the advantage of linezolid over vancomycin in the therapy of staphylococci-based nosocomial-acquired pneumonia [37]. However, other studies reported no decisive benefit of linezolid over vancomycin for treating MRSA nosocomial-acquired pneumonia [38,39]. There are still different recommendations for the management of MRSA pneumonia. After considering the effectiveness and cost-efficiency, vancomycin was recommended as the first antibiotic of choice for most inpatients suffering nosocomial-acquired pneumonia by MRSA. Linezolid was recommended as a meaningful alternative for inpatients in whom vancomycin failed [40]. It is not yet clear which treatment option should be considered the best medication and first selection for the management of MRSA pneumonia. Another study showed a similar effectiveness of linezolid compared to vancomycin, and neither of these antibiotic drugs was superior for treating MRSA nosocomial-acquired pneumonia [41]. Although the MIC breakpoints for linezolid in this study did not change after the switch to the EUCAST guidelines, an elevation trend of susceptibility was noticed for linezolid with increasing numbers of cases of pneumonia due to *S*. *aureus* and MRSA in the past several years. It should be noted that linezolid was tested in a low number of patients, primarily on the isolates of *S*. *aureus* in this study.

Another recommendation for the antibiotic therapy of MRSA pneumonia is the administration of teicoplanin [42]. Teicoplanin is employed in pharmacological treatment against a range of bacterial infections, particularly Gram-positive pathogens [43]. Although resistance to teicoplanin has been reported [44], *S*. *aureus* and MRSA did not develop antibiotic resistance toward this antibiotic in the present investigation. However, all of the *S*. *aureus* and MRSA isolates were susceptible to teicoplanin apart from the decreased frequency of susceptibly testing.

Meanwhile, both the evolution and the utilization of antibiotics for the management of bacterial infectious diseases are becoming more difficult and expensive due to the spread of the clinically problematic mechanisms of antibiotic resistance. When a new drug is brought to market after years of development, it takes only a short time for microorganisms to become resistant to it [45]. Recently, tetracycline was added to clinical applications. Tetracycline is an antibiotic with broad-spectrum activity and it is used against many bacterial infections [46], and it is well known that it is highly active against resistant bacteria. Unfortunately, there is already a bacterial-resistance mechanism even for tetracycline, which has caused it to no longer play a role in the application of medicine. It is feared that this resistance mechanism of tetracycline will spread to MRSA [47]. The development of MRSA resistance toward tetracycline was observed in one isolate in the current study.

Rifampicin was reported to increase the effect on *S*. *aureus* in combination with vancomycin through better tissue penetration [48,49]. A number of reports have also found an improvement in the effects against MRSA through the combination therapy of vancomycin and rifampicin [50]. Although rifampicin has a number of advantageous properties as an adjunctive agent with vancomycin, the results obtained in studies were often contradictory, and there are clinical trial results that do not support the use of rifampicin in co-administration with vancomycin [51]. The present study found rifampicin resistance in MRSA pneumonia. These findings support the recommendation that the treatment of MRSA pneumonia with rifampicin should be monitored to detect the development of rifampicin resistance.

Co-trimoxazole is an antibiotic composed of trimethoprim and sulfamethoxazole, used to kill Gram-positive and Gram-negative bacteria. One study evaluated the efficacy of co-trimoxazole in comparison with vancomycin, and the investigation showed that co-trimoxazole had the same effect as vancomycin in the management of nosocomial-acquired pneumonia triggered by MRSA [52]. However, a different trial reported that vancomycin had better efficacy and safety than co-trimoxazole in the management of staphylococcal pneumonia [53]. In the current study, only *S*. *aureus* showed an elevated resistance rate against co-trimoxazole, while good sensitivity was similarly detectable.

Other antibiotics, such as fosfomycin, are indicated to treat severe staphylococcal infections [54]. While resistance to fosfomycin was discovered in this study, a past study recommended fosfomycin in combination with vancomycin or linezolid [55].

The antibiotic gentamicin acts mainly against Gram-negative bacteria but also shows activity against staphylococci [56]. A previous study reported especially good results for aerosolized gentamicin in the treatment of staphylococcal pneumonia [57]. Resistance of staphylococci to gentamicin is known, and several years ago an outbreak of gentamicin-resistant MRSA pneumonia was reported [58]. Gentamicin-resistant MRSA was found in two MRSA cases and eight *S*. *aureus* cases in this study. The antibiotic resistance rate of gentamicin in *S*. *aureus* was about 5.5% in this study, similar to the outcome of another investigation [59]. Therefore, a combination therapy of gentamicin and vancomycin in the management of MRSA pneumonia is often recommended.

Cefepime has been described as having good activity against *S*. *aureus* [60]. This antibiotic has shown effectiveness towards many Gram-negative and some Gram-positive microorganisms [61]. Cefepime resistance was found in staphylococcal isolates of inpatients with pneumonia in the current investigation.

The antibacterial spectrum of tobramycin is quite wide, from Gram-negative bacteria to Gram-positive cocci, for example *S*. *aureus* [62]. Antibiotic resistance to tobramycin was detected both in this study and in an earlier study [63]. Tobramycin has proven to be effective through inhalation treatments for pneumonia [64].

Fusidic acid is a steroid antibiotic isolated from fungi. The spectrum of activity of fusidic acid is small, and it is mainly active on Gram-positive pathogens such as staphylococci [65]. All MRSA infections were susceptible to fusidic acid in nosocomial-acquired pneumonia in a past study [66], as well as in the current study. Staphylococci are known to have antimicrobial resistance to fusidic acid [67], and this was also found in *S*. *aureus* in the present investigation.

Although cefotaxime has a broad spectrum of effectiveness, it has insufficient efficacy against staphylococci [68–70]. This lack of effectiveness against *S*. *aureus* and MRSA was also observed in the current study.

Representatives from other classes of antibiotics, such as levofloxacin, are somewhat effective against *Staphylococcus*, but rapid resistance is developing [71,72]. The low efficacy of levofloxacin on *S*. *aureus* and MRSA was also demonstrated in the present study.

Similarly, cephazolin revealed elevated antibiotic resistance in *S*. *aureus* and MRSA in this study. This was comparable to the elevated antibiotic resistance of staphylococci to cephazolin correspondingly reported in a previous study [73]. Cephazolin is effective only against certain pathogens, such as staphylococci, among other microbes [74].

Furthermore, it should be noted that while clindamycin is effective against staphylococci, increased antibiotic resistance in *S*. *aureus* and MRSA to clindamycin was noted in the present study. The development of antibiotic resistance of staphylococci to clindamycin has been increasingly observed in past studies [75,76].

While cefuroxime has a good effect even against staphylococci due to its vast antimicrobial activity spectrum, *S*. *aureus* and MRSA showed increased resistance to this antibiotic in this investigation. These findings of raised cefuroxime resistance in staphylococci were previously reported in other studies [77,78].

Similar unfavorable results were obtained for the antibiotic erythromycin in this investigation, which found increased antibiotic resistance in *S*. *aureus* and MRSA toward erythromycin. Erythromycin is effective against a variety of Gram-positive bacteria; its effectiveness against staphylococci may be reduced by the development of antibiotic resistance [79].

Ultimately, the worst outcomes in this study were observed for the antibiotic ciprofloxacin, which mainly has antimicrobial effectiveness against Gram-negative bacteria and only a small effect against Gram-positive bacteria such as staphylococci [80]. Therefore, as is already known, ciprofloxacin is not suggested for the management of staphylococcal pneumonia [81].

In this study, even more remarkable was the growing antibiotic resistance of staphylococci to penicillin and ß-lactamase inhibitor antibiotics. Staphylococci are generally sensitive to ß-lactam antibiotics [82]; however, a large proportion of staphylococcal strains are developing antibiotic resistance to penicillin [83]. This antibiotic resistance is based on the formation of penicillinases [84,85]. Therefore, various penicillin antibiotics are administered in mixtures with lactamase inhibitors, such as sulbactam and tazobactam [86]. More germs are continually becoming resistant to penicillin and to lactamase inhibitors, not only due to natural mutation and selection but also due to the somewhat irresponsible frequency with which penicillin and lactamase inhibitors are utilized [87].

### Study limitations

The findings of this study are unique to a large university hospital and therefore the conclusions drawn from this report cannot be generalized to other clinics. Also, after the evaluation of the susceptibility tests, it had to be noted that not all antimicrobial agents were used with the same frequency in the performance of susceptibility tests of patients with *S*. *aureus* and MRSA pneumonia.

## Conclusions

Current therapy options for MRSA pneumonia are limited. The emergence of resistance to newer antimicrobial agents, including linezolid, vancomycin, and teicoplanin, has been reported for *S*. *aureus*. However, in the current study, the *S*. *aureus* isolates were susceptible to all of these antibiotics, although they developed resistance to a variety of other antibiotics. We conclude that two antimicrobial agents, linezolid and vancomycin, may be considered in MRSA-associated pneumonia, with serum-level monitoring and in combination with rifampicin to augment the synergistic effect. For methicillin-susceptible *S*. *aureus* infections, fluoxacillin is a therapy option for pneumonia patients. However, more clinical studies are needed to better define the role of linezolid in MRSA pneumonia and MRSA sepsis, for instance in different patient populations, e.g. COPD or otherwise immunocompromised patients. Further research is also needed on the cost-effectiveness of linezolid and the outcome measurements and hospital length of stay in head-to-head trials versus vancomycin.

## Abbreviations

ANOVA: analysis of variance
CLSI: Clinical and Laboratory Standards Institute
CRP: C-reactive protein
CI: confidence interval
EUCAST: European Committee on Antimicrobial Susceptibility Testing
ICD: International Classification of Diseases
MIC: minimum inhibitory concentration
MRSA: methicillin-resistant *Staphylococcus aureus*
